# Clinical and patient reported outcomes in breast reconstruction using acellular dermal matrix

**DOI:** 10.1016/j.jpra.2018.06.006

**Published:** 2018-07-05

**Authors:** S. Powell-Brett, S. Goh

**Affiliations:** The Breast Unit, Peterborough City Hospital, Edith Cavell Campus, Bretton Gate, Peterborough, PE3 9GZ, England

**Keywords:** ADM, Breast reconstruction, Mastectomy, Breast cancer

## Abstract

**Introduction:**

There is a lack of published patient reported outcome measures (PROMs) for the use of acellular dermal matrix (ADM) based breast reconstruction. This cohort study reviewed our clinical outcomes and PROMs.

**Methods:**

All patients undergoing mastectomy with ADM assisted immediate breast reconstruction under a single surgeon between June 2013 and June 2017 were included. A prospectively kept database, clinic letters and operation notes were analysed. All patients received BREAST-Q^TM^ pre and post-operative questionnaires.

**Results:**

Sixty-two consecutive patients with 77 reconstructions were included. Mean hospital stay was 3.3 days. All patients received 48 h of intravenous antibiotics, followed by a two-week course of oral antibiotics. Mean post-operative follow up was 17 months. There were 8 cases of skin necrosis (10.4%), and 1 infection (1.3%). These resulted in 4 explantations (5.2%); 3 following skin necrosis and 1 following infection. There was no observed ‘red skin’ syndrome. Post-operative mean score for ‘satisfaction with outcome’ was 83.1%. Mean score for ‘Psychosocial well-being’ was 70.7% and the mean score for ‘physical well-being’ was 77.9%.

**Conclusion:**

Our complication rates were comparable to those published, and PROMs were consistently good. The skin necrosis rate was potentially due to earlier practice of performing single-stage immediate reconstruction using fixed volume breast implants. We have modified our patient selection criteria and ADM based reconstructive techniques with experience. Longer term clinical and patient reported outcome should be sought.

## Introduction

Breast cancer represents the commonest malignancy in the UK, with around 55,200 new cases in 2014, accounting for 31% of cancers in women.^1^ Mastectomy and immediate reconstruction is now commonplace; just under half of women undergoing surgery for breast cancer have a mastectomy and a third of those undergoing mastectomy have an immediate reconstruction.^2^, ^3^

Options for immediate reconstruction remain varied but implant based reconstruction remains the most popular with 37% of immediate breast reconstructions in the UK being implant based.^3^ The use of acellular dermal matrix (ADM) has been gaining popularity for its use in ameliorating some of the aesthetic challenges faced with implant based reconstruction. ADM provides a scaffold upon which the patient's own cells may repopulate and vascularize, allowing breast surgeons a means by which to cover an implant with vascularised soft tissue. In addition, ADM enables better definition of the inframammary fold and a more natural projection and ptosis. Statistics for the use of ADM in the UK are hard to quantify but in America it is used in over half of implant based reconstruction.^4^ The literature surrounding ADM in breast reconstruction though becoming more commonplace is still difficult to analyse. Recent reviews have been unable to come to conclusive statements on its safety and benefits owing to a lack of comparable data and the wide variety of products on the market. A review in 2017 found only twelve studies comparing ADM to no ADM, one of which was prospective and randomised but encompassed very small numbers. The same review also found only 10 studies looking at post-operative complications with ADM (no comparator group), only 3 of which were prospective. Of those studies looking at post-operative complications, explantation rates ranged from 0% to 11%. The wide range of published complication rates and limited published high-grade evidence makes it difficult to come to firm recommendations of its use.

Breast surgery is fundamentally deforming and can have negative effects on body image and self-esteem which can result in depression, anxiety, shame and even suicide.^5^ Patient's own perceptions of the impact of breast cancer and surgical treatment are increasingly being recognised as fundamental to understanding overall health outcomes.^6^, ^7^ It is thus imperative to use external methods of assessment of quality of life in the form of Patient Reported Outcome Measures (PROMs). The BREAST-Q is an independently validated scoring system for collecting patient reported outcome measures and has a specific module for breast reconstruction.^8^ The BREAST-Q examines two domains; patient satisfaction and patient quality of life. Under these two domains are six subthemes (Physical, Psychosocial and Sexual well-being under quality of life; Satisfaction with breasts, Satisfaction with overall outcome and Satisfaction with Care under patient satisfaction).^9^

The aims of this study are firstly to examine our own surgical outcomes and secondly to collect PROMs data in order to fully assess our units’ quality of care and to add to the growing body of evidence on the use of ADM.

## Method

This prospective cohort study recruited 62 consecutive patients with 77 reconstructions from June 2013 to June 2017. Included were all patients who underwent mastectomy and immediate reconstruction with implant and ADM under a single surgeon. All patients underwent consultation with both an oncoplastic trained surgeon and specialist breast care nurse to discuss the full range of reconstructive options before deciding on this form of reconstruction. There were no exclusion criteria and no pre-selection. All patients were discussed at breast MDT and underwent adjuvant and neo-adjuvant therapy as advised. The choice of reconstructive procedure had no impact on this decision or the timing of this treatment. At pre-operative consultation, advice was given to smokers on the advantages of quitting smoking pre-operatively but smokers were not selected against. With permission, our unit utilised the BREAST-Q reconstruction module and the registered BREAST-Q scoring software to analyse the results. At a set time point all patients were invited to fill in pre-operative and post-operative BREAST-Q PROMs questionnaires via post.

Data collected included basic patient demographics, smoking status, BMI, neo-adjuvant treatment, adjuvant treatment, tumour characteristics, length of stay, antibiotic duration, drain duration, post-operative contact, post-operative complications, further interventions and final follow up date. A database was kept prospectively and data was collected via electronic notes, MDT records and operation notes.

Patient care was administered as standard for that operating surgeon and a consistent, reproducible approach was taken. All patients were admitted on the day of surgery, received 1.2g co-amoxiclav at induction, 48 h of IV antibiotics (1.2g TDS) and two weeks of oral co-amoxiclav (625 mg TDS). A single surgeon performed all procedures with a standardised technique, although experience led to some minor adjustments. A nipple sacrificing mastectomy using sharp dissection was performed on all. The implant was placed in the sub-pectoral plane with the ADM (Strattice^TM^) fixed inferiorly using 2.0 PDS and along the inferior border of pectoralis major using 2.0 monocryl. The ADM was fenestrated with a 4mm punch biopsy needle at 10–15 places. 2 drains were placed outside of the ADM/pectoral pocket, one superior and one inferior (in front of the ADM). One adjustment was the reduction in the use of large (more than 550cc), fixed volume implants in favour of the use of expanders in cases where the mastectomy pocket was felt to be under tension with a fixed volume implant of the most appropriate size for the patient. Where a fixed volume implant was felt appropriate, Mentor CPG ® anatomical implants were used, and where an expander was needed, Becker-35 ® expandable implants were used. All patients had surgical follow up at a week (with removal of the superiorly placed drain) and two weeks (with the removal of the inferiorly placed drain) post-operatively and thereafter as required.

## Results

Sixty-two consecutive patients with 77 reconstructions were included in this study. Fifty-eight mastectomies were performed for the treatment of cancer and 19 were performed for prophylaxis. Patient demographics are shown in Table 1 and tumour biology in Table 2.

**Table 1:** Patient demographics.

***Mean Age***	*49 years*
***Mean BMI***	*25.1*
***Smoker***	*9 (11.7%)*
***Unilateral***	*47*
***Bilateral***	*15*
***Pre-op radiotherapy***	*13 (16.9%)*
***Post-op radiotherapy***	*19 (24.7%*

**Table 2:** Tumour biology.

***IDC***	*37*
***ILC***	*7*
***LCIS***	*1*
***DCIS***	*10*
***Pagets***	*1*
***papillary***	*1*
***metaplastic***	*1*
***N/A***	*19*

### Post-operative complications

All patients were seen by a breast surgeon post-operatively and on at least three separate occasions in outpatient clinic. Follow up ranged from five weeks to four years, with a mean follow up time of eighteen months. The most common complication was skin flap necrosis, eight cases were (10.4%) identified, three resulted in implant loss, two underwent superficial debridement only and three required no surgical intervention. There were four haematomas identified (5.2%), only one of which required evacuation. There was a single case of seroma (1.3%) that did not require drainage and a single case of infection (1.3%) that did lead to implant loss. Overall there were four cases requiring implant removal (5.2%), one following infection and three following skin necrosis. (Table 3).

**Table 3:** Post-operative complications.

			NMBRA outcome	NMBRA target
***Implant loss***		*4 (5.2%)*	*9%*	*<5%*
***Antibiotics at three months***		*1 (1.3%)*	*25%*	*<10%*
***Return to theatre for local complications***		*5 (6.4%)*	*7.6%*	*<5%*
***Infection***		*1 (1.3%)*		
***Skin necrosis***	***Total***	***8 (10.4%)***		
	*Conservative management*	*3 (3.9%)*		
	*Superficial debridement only*	*2 (2.6%)*		
	*Leading to implant loss*	*3 (3.9%)*		
***Haematoma***	***Total***	***4 (5.2%)***		
	*Conservative management*	*3 (3.9%)*		
	*Evacuation*	*1 (1.3%)*		
***Seroma***	***Total***	***1 (1.3%)***		
	*Requiring drainage*	*0*		

### Patient reported outcome measures

PROMs outcomes were analysed via the registered PROMs Breast-Q scoring system and results are given as a score out of one hundred for each of the domains. Results are presented below as the mean score for each domain and the respective standard deviation. (Table 4).

**Table 4:** Mean Breast-Q score at 6 months post-operatively (Mean +/− SD).

**Satisfaction with**	Outcome	**82.5 +/**−**18.9**
	Breasts	**67 +/**−**19.1**
	Information	**90.7 +/**−**15.1**
**Satisfaction with**	Surgeon	**97.4 +/**−**11.7**
	Medical team	**96.1 +/**−**10.4**
	Office staff	**89.7 +/**−**22.1**
**Well being**	Psychosocial	**71.3 +/**−**20.5**
	Sexual	**55.9 +/**−**20.4**
	Physical	**77.9 +/**−**19.2**

**Image 1:**
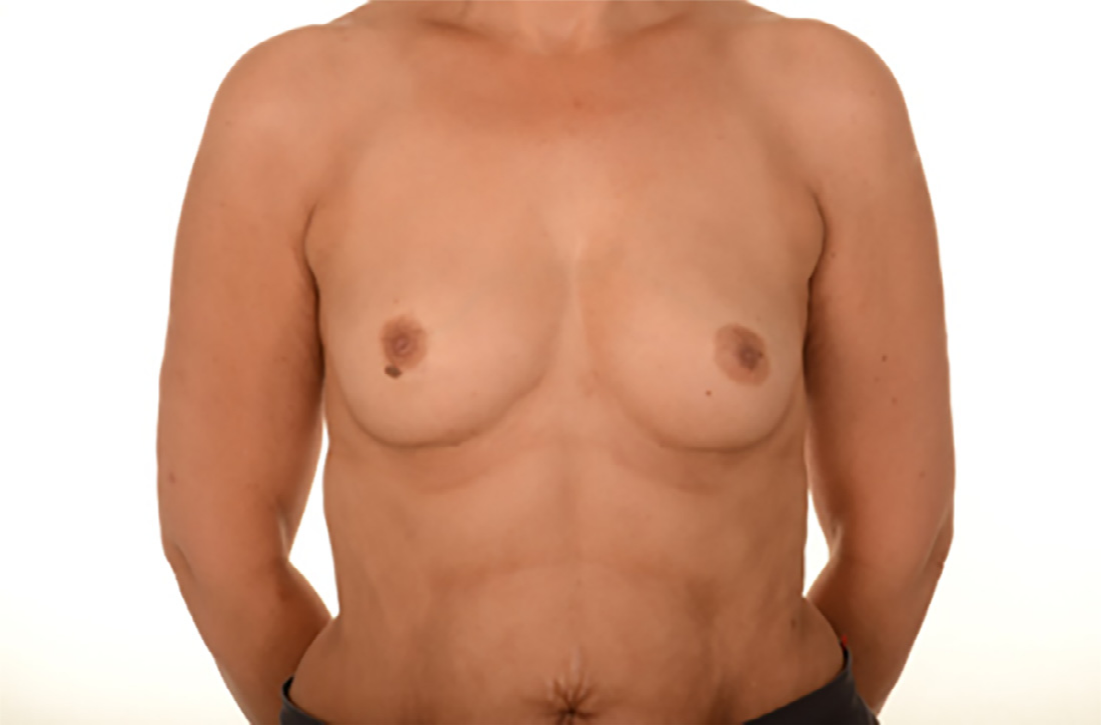
Patient A: 54 year old, Pre-operative.

**Image 2:**
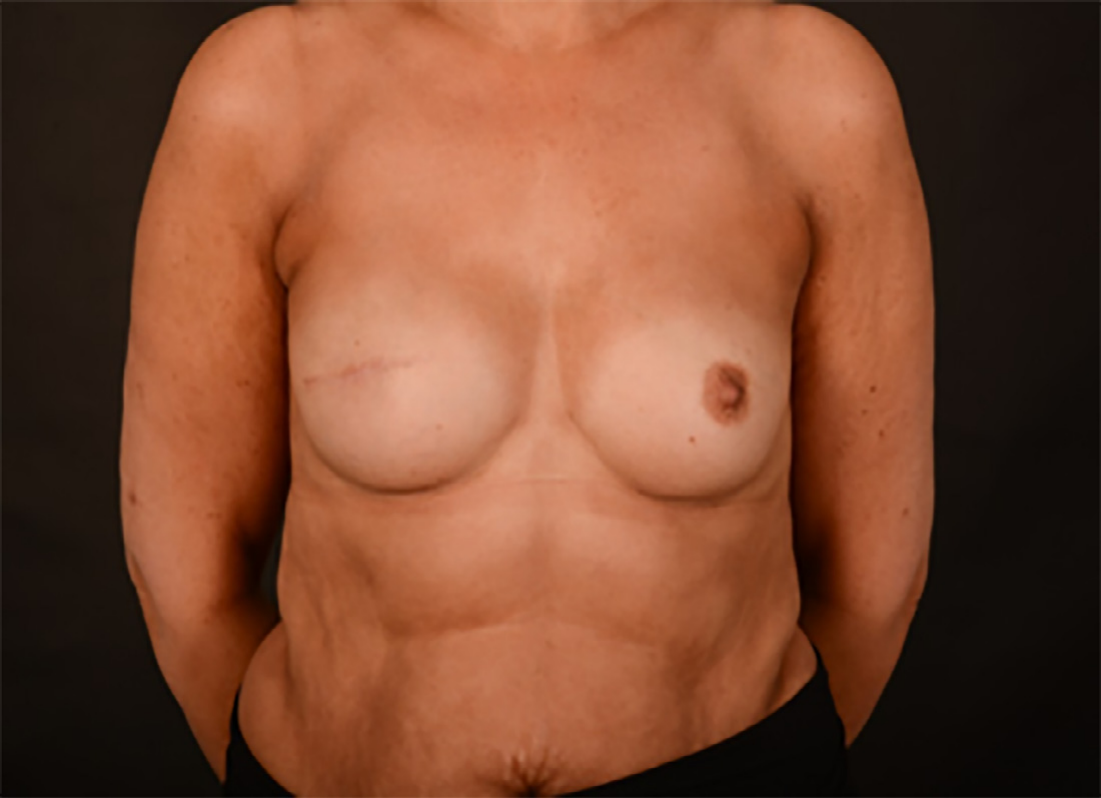
Patient A: Three months post-operative.

**Image 3:**
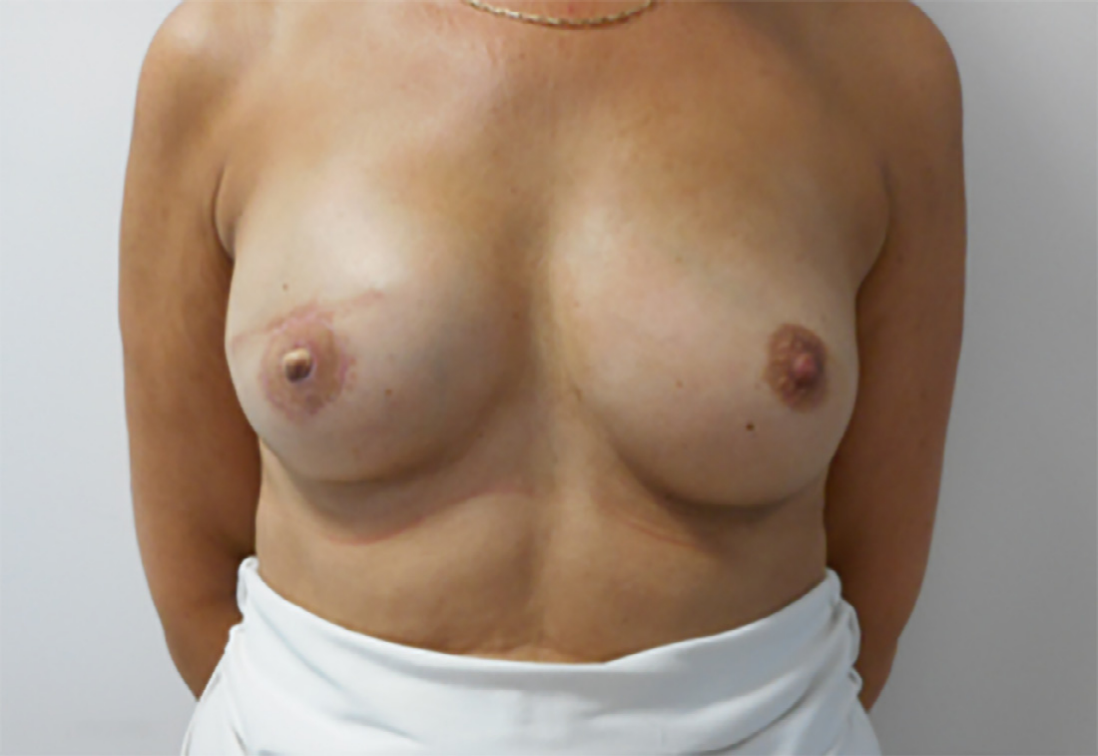
Patient A: Twelve months post-operative with nipple areola reconstruction.

**Image 4:**
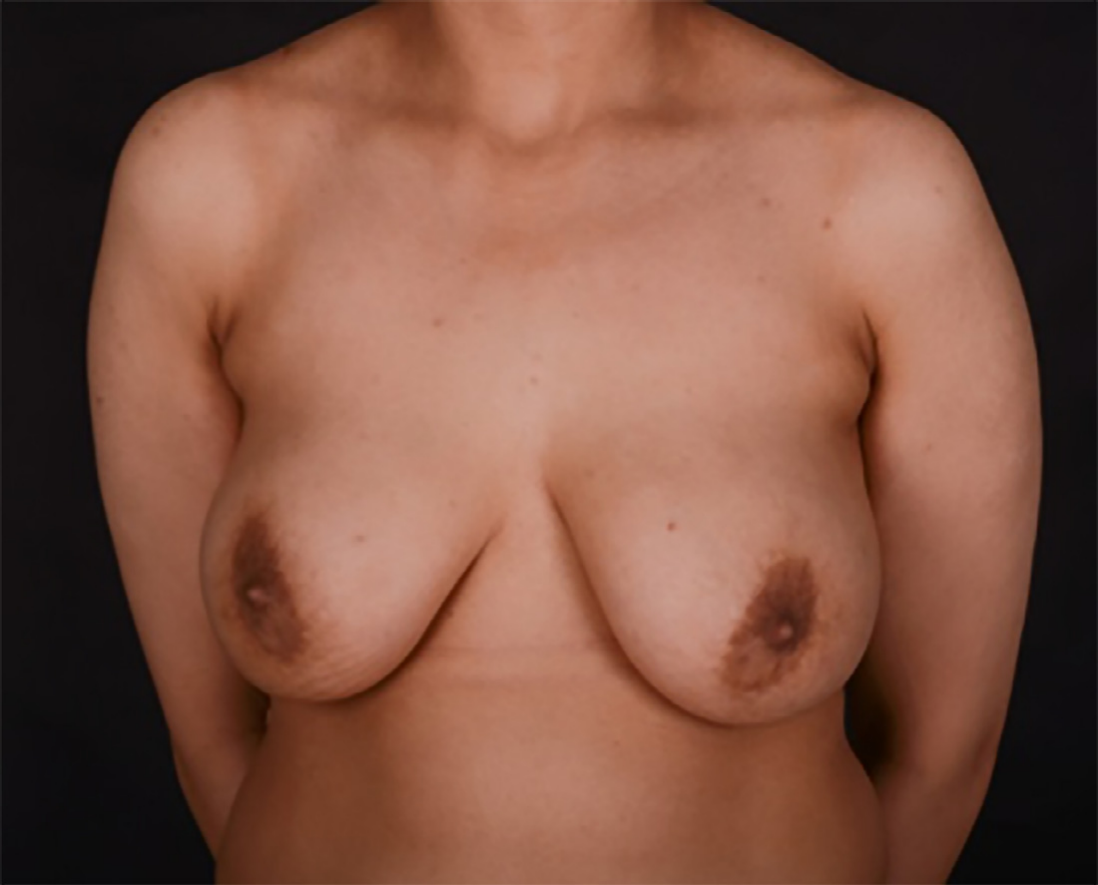
Patient B: 31 year old, bilateral simultaneous cancers, pre-operative.

**Image 5:**
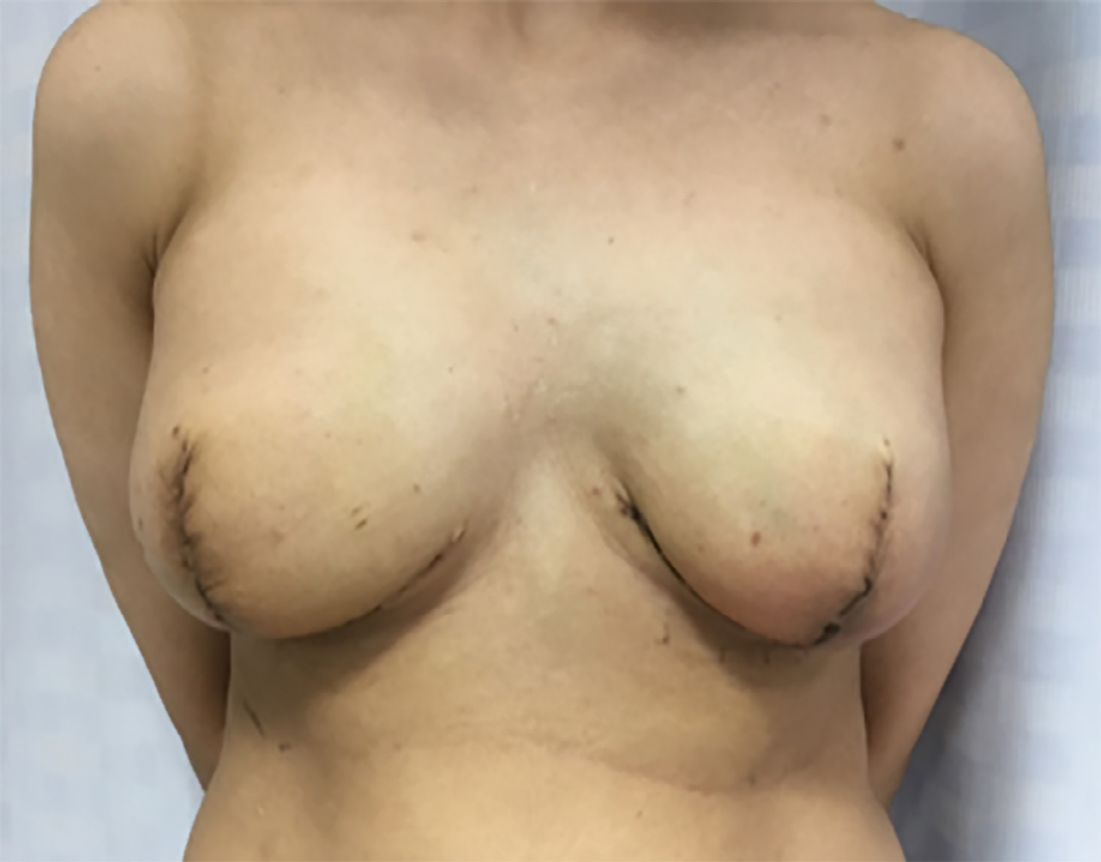
Patient B: One month post-operative.

Pre- and post-operative photos were obtained where possible and with written consent. Images 1–5 shows two patients having undergone implant based reconstruction using ADM, patient A (Images 1–3) had a unilateral mastectomy and reconstruction with subsequent nipple reconstruction and patient B (Images 4 and 5) had bilateral mastectomy and reconstruction without nipple reconstruction.

## Discussion

### Post-operative complications

Since their introduction for use in burns victims in the 1990s the use of ADM has been increasing, the first published use of ADM for breast reconstruction was in 2001 to correct implant rippling [10]. Since then the published literature on outcomes with ADM assisted reconstruction has been inconsistent and scanty. A major confounding factor is the variety of products currently on the market. The majority of early studies concluded that the use of ADM was associated with inferior outcomes, mainly based on increased rates of infection and implant loss. (11) However in 2012, a systematic review published by Macadam and Lennox found that in single stage, direct to implant reconstruction, the use of ADM resulted in reduced rates of infection, seroma and implant loss. However, Macadam and Lennox still found a higher risk of skin necrosis with the use of ADM. A meta-analysis by Ho et al. found skin flap necrosis as the most common complication associated with ADM assisted reconstruction, standing at 10.9% with high rates of seroma (6.9%), infection (5.7%) and implant loss (5.1%). Our results are comparable to those published in these meta-analyses with the main complication being skin necrosis. The impact of this is difficult to quantify as although the rate of skin necrosis in our study was 10.4%, less than half of these resulted in reconstructive failure and a quarter of them were managed conservatively with complete resolution. Our results also reflect the emerging evidence that single stage implant based reconstruction with ADM shows favourable results when looking at rates of seroma and infection (in contrast to some of the earlier published literature) with only a single case of infection and a single seroma (that did not require drainage). Comparing our units outcomes to the National Mastectomy and Breast Reconstruction Audits's (NMBRA) outcomes and targets (as used in the joint guidelines from the Association of Breast Surgery (ABS) and the British Association of Plastic, Reconstructive and Aesthetic Surgeons (BAPRAS)), these results are below NMBRA's outcomes and in line with their targets, except for the rate of return to theatre for local complications which was 6.4% where the target is <5% and NMBRa's outcome was 7.6%.

### Patient reported outcome measures

In the wake of a cosmetically deforming operation that is well recognised to have potential negative effects on body image and self-esteem, patient satisfaction and quality of life must be recognised as a significant outcome when evaluating surgical success. Unfortunately, PROMs data has no standardised format and is inconsistently reported. This makes it difficult to accurately make comparisons and parallels between different publications. Even without comparison, our units results are excellent with a mean score of 82.5/100 for overall ‘satisfaction with outcome’, a mean score of 71.3/100 for ‘Psychosocial well-being’ and a mean score of 77.9/100 ‘physical well-being’. These compare favourably in all domains when compared to a similar study published in 2016 by Vu et al. that collected PROMs data for 72 reconstruction using ADM. However, despite a lack of comparative data within ADM based reconstruction, the information gleaned from PROMs is important in enabling patients to make an informed decision about the type of breast reconstruction they wish to undergo. With PROMs data becoming more widely collected across the different reconstructive techniques this should become increasingly possible

## Conclusion

This study reflects high levels of satisfaction across most domains of the BREAST-Q, especially with regard to overall satisfaction. Minimal problems with infection, seroma and haematoma are re-assuring, however the relatively high rates of skin flap necrosis, (although still in line with the wider literature and below the outcomes of the NMBRA audit) is still of concern. An evolving surgical technique to prevent undue thinning of and tension across the skin flaps may help to bring this figure down. In an era of evolving use of ADM it is imperative to ensure the continued evaluation of outcomes, including prospective PROMs data collection to enable comparison of different techniques and the maintenance of high standards.

## Conflict of interest

None

## Funding

None
